# Supplementary figures and images for: Metabolites profiling of Sapota fruit pulp via a multiplex approach of gas and ultra performance liquid chromatography/mass spectroscopy in relation to its lipase inhibition effect
Source: PeerJ. 2024 Aug 29;12:e17914. doi: 10.7717/peerj.17914 (PMC11366232; doi:10.7717/peerj.17914)

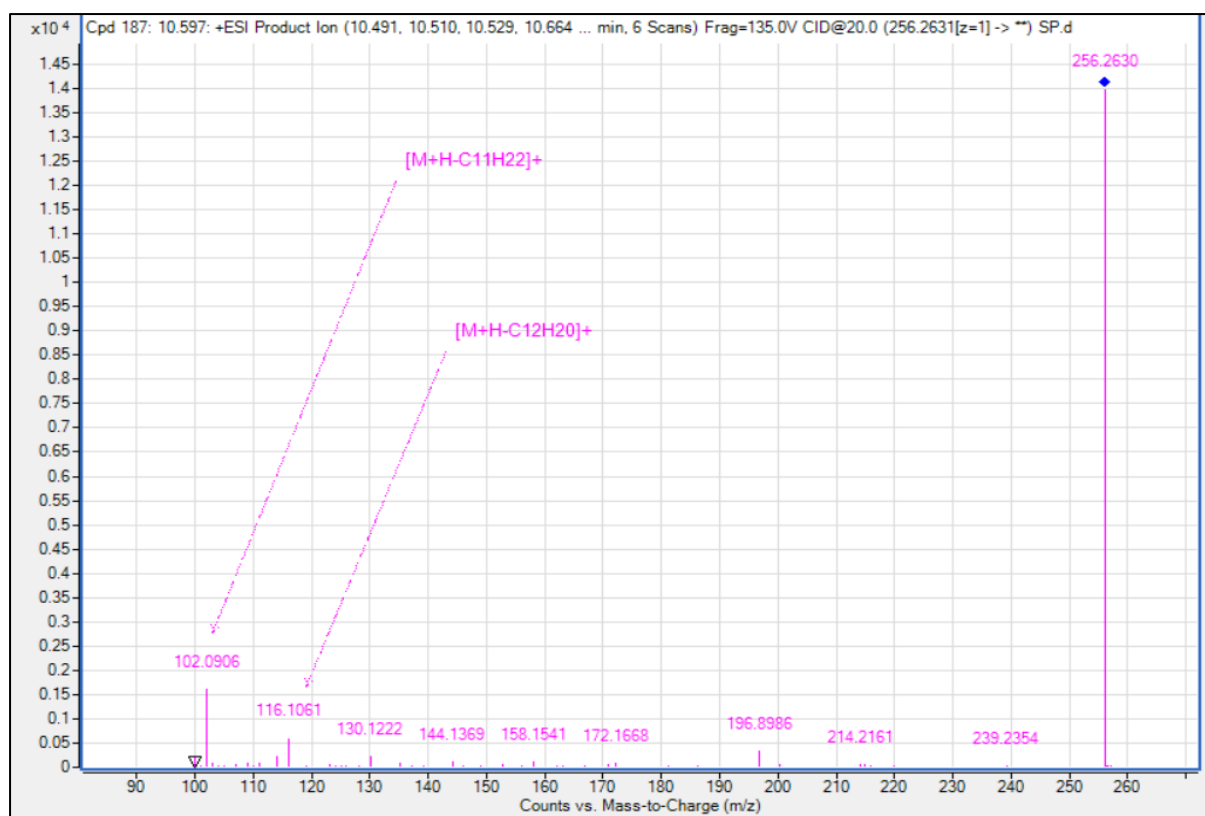

Supplement: Supplemental Information 1 [file peerj-12-17914-s001.pdf]

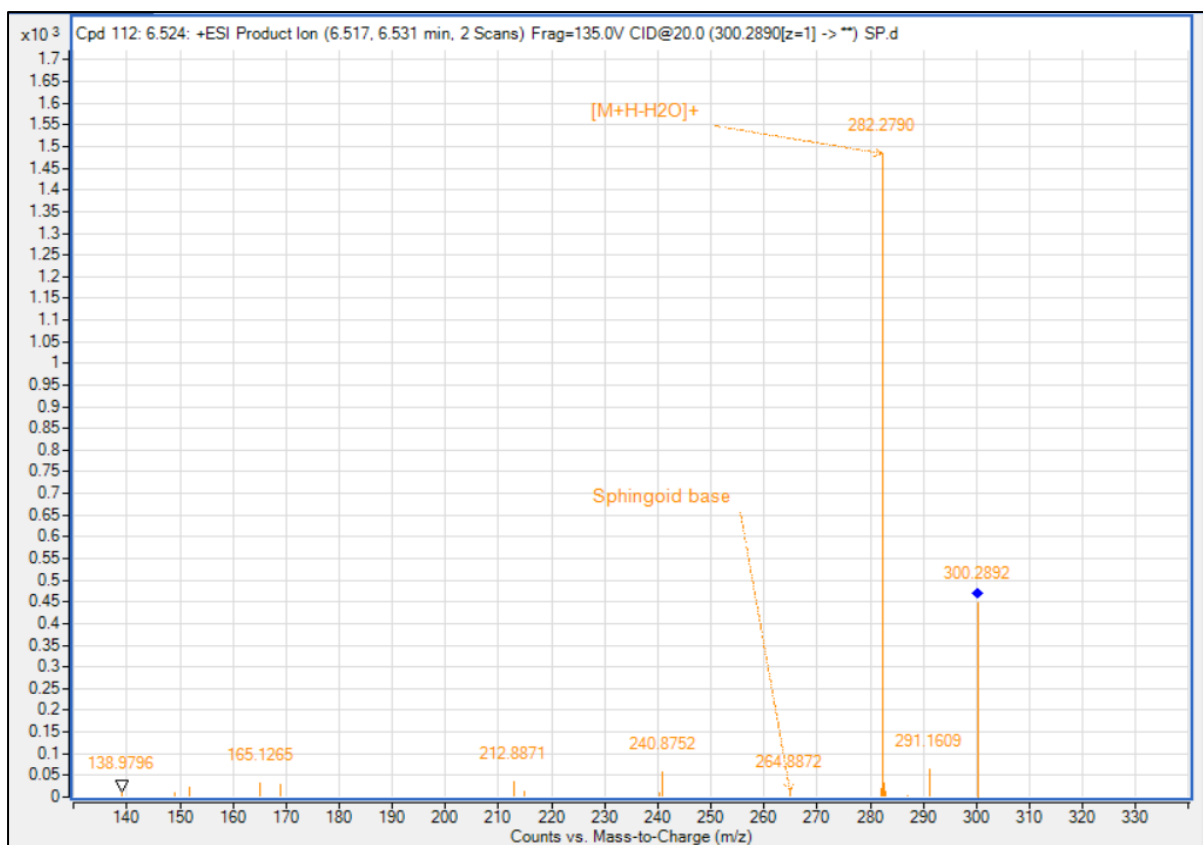

Supplement: Supplemental Information 2 [file peerj-12-17914-s002.pdf]

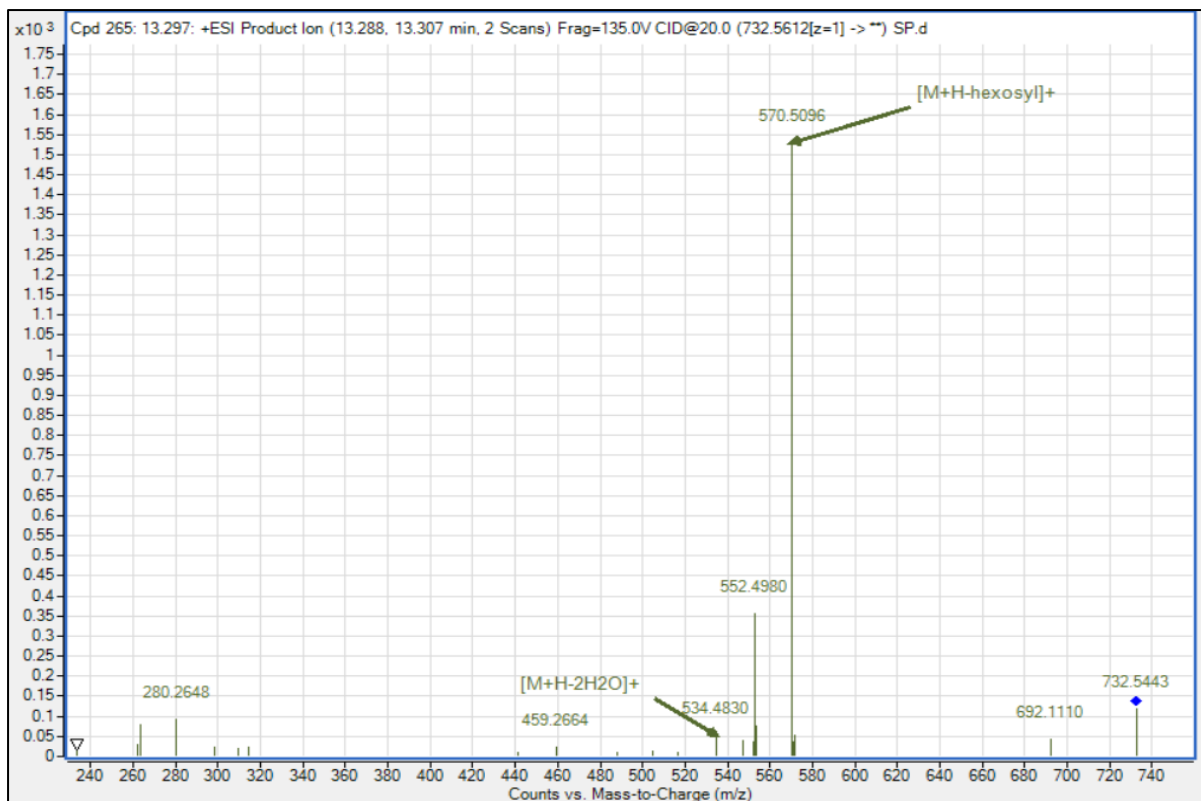

Supplement: Supplemental Information 3 [file peerj-12-17914-s003.pdf]

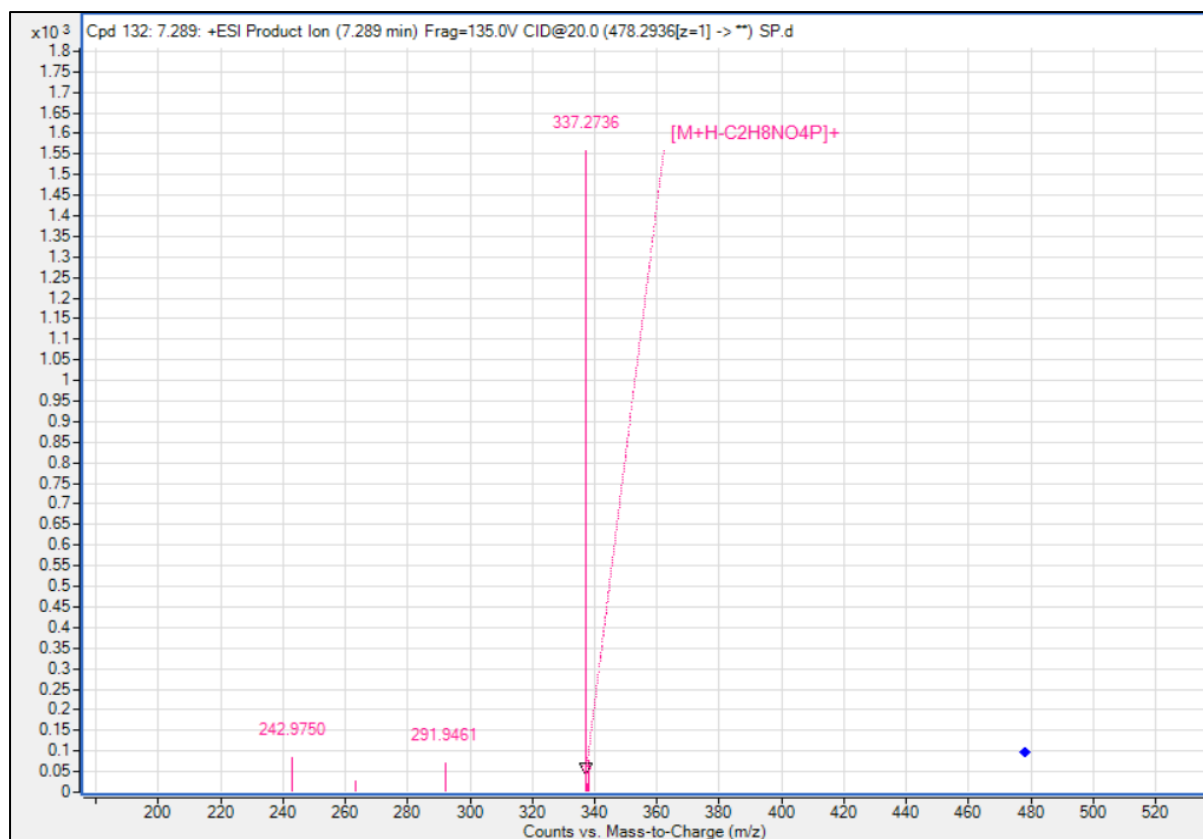

Supplement: Supplemental Information 4 [file peerj-12-17914-s004.pdf]

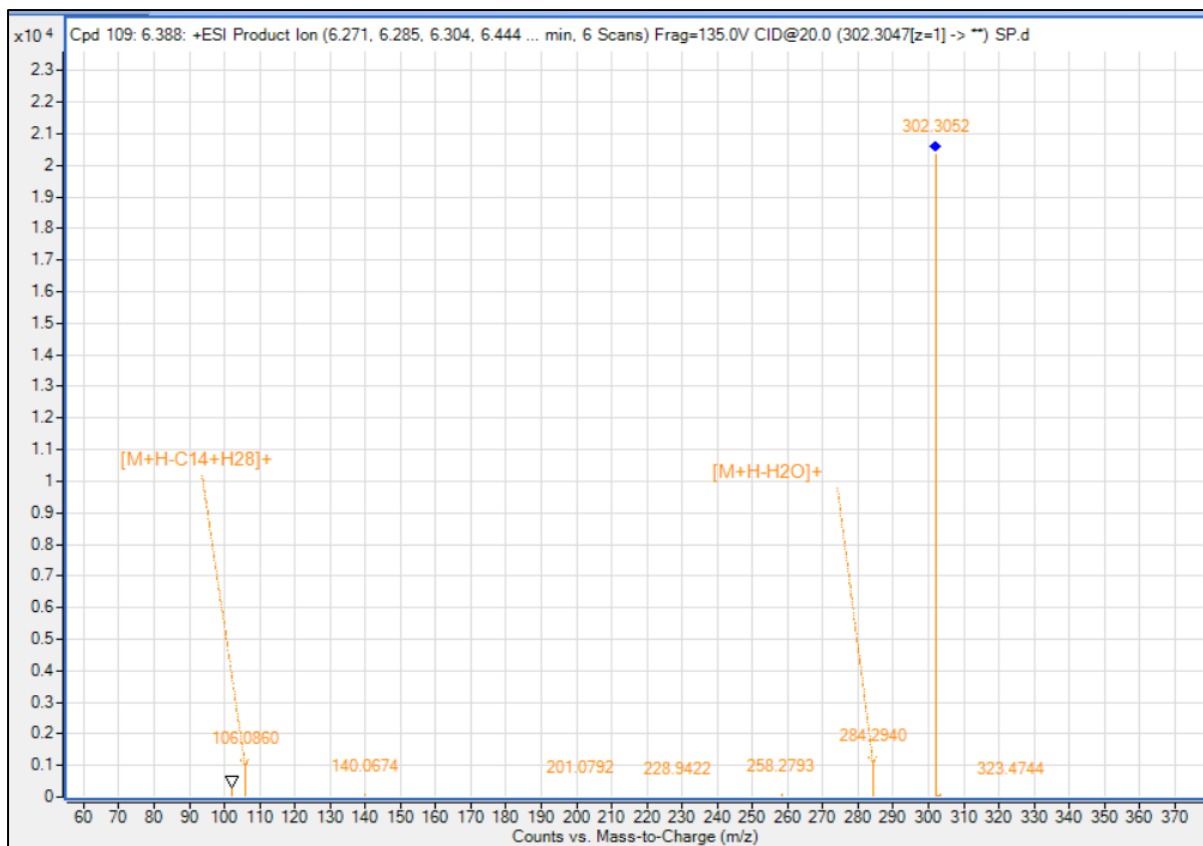

Supplement: Supplemental Information 5 [file peerj-12-17914-s005.pdf]

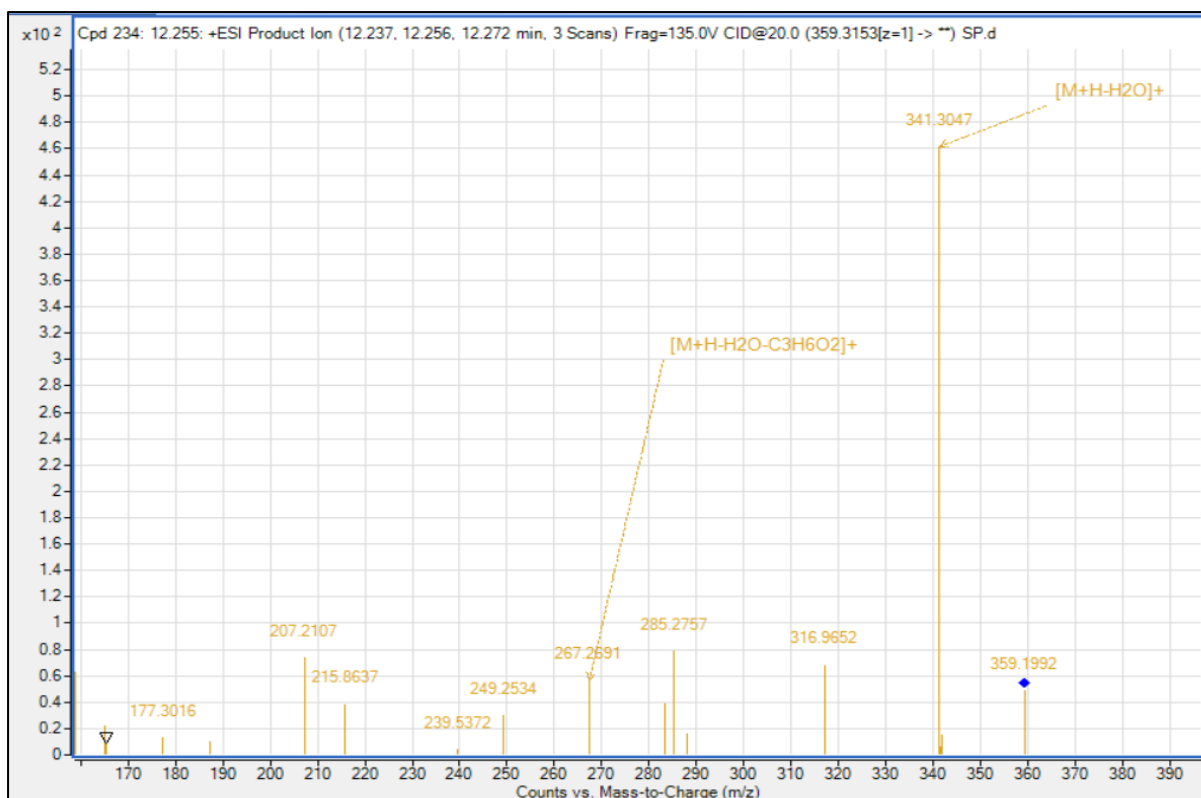

Supplement: Supplemental Information 6 [file peerj-12-17914-s006.pdf]

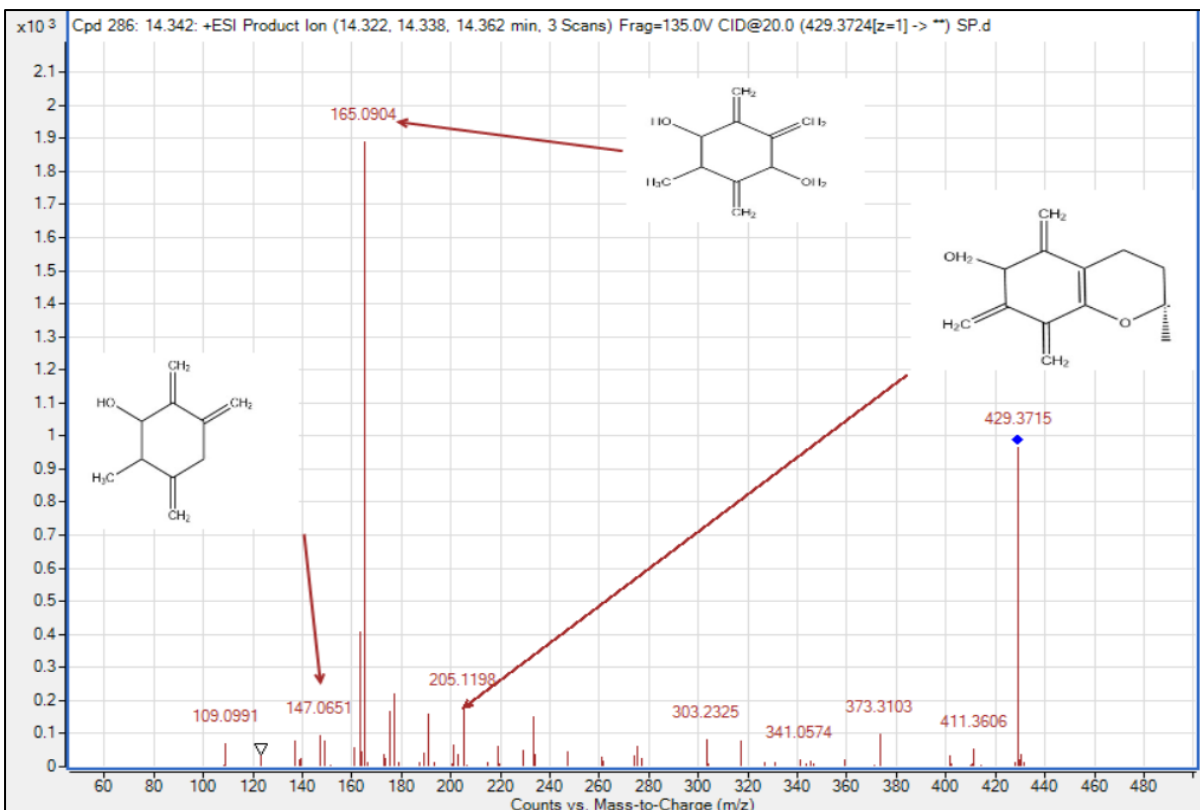

Supplement: Supplemental Information 7 [file peerj-12-17914-s007.pdf]
